# Integrating data from an online diabetes prevention program into an electronic health record and clinical workflow, a design phase usability study

**DOI:** 10.1186/s12911-016-0328-x

**Published:** 2016-07-11

**Authors:** Rebecca Grochow Mishuris, Jordan Yoder, Dan Wilson, Devin Mann

**Affiliations:** Boston University School of Medicine, 801 Massachusetts Avenue, Crosstown 2nd floor, Boston, MA 02118 USA; Moxe Health, Madison, Wisconsin USA

**Keywords:** Clinical decision support, Electronic health record, Usability testing, e-health, Preventive medicine

## Abstract

**Background:**

Health information is increasingly being digitally stored and exchanged. The public is regularly collecting and storing health-related data on their own electronic devices and in the cloud. Diabetes prevention is an increasingly important preventive health measure, and diet and exercise are key components of this. Patients are turning to online programs to help them lose weight. Despite primary care physicians being important in patients’ weight loss success, there is no exchange of information between the primary care provider (PCP) and these online weight loss programs. There is an emerging opportunity to integrate this data directly into the electronic health record (EHR), but little is known about what information to share or how to share it most effectively. This study aims to characterize the preferences of providers concerning the integration of externally generated lifestyle modification data into a primary care EHR workflow.

**Methods:**

We performed a qualitative study using two rounds of semi-structured interviews with primary care providers. We used an iterative design process involving primary care providers, health information technology software developers and health services researchers to develop the interface.

**Results:**

Using grounded-theory thematic analysis 4 themes emerged from the interviews: 1) barriers to establishing healthy lifestyles, 2) features of a lifestyle modification program, 3) reporting of outcomes to the primary care provider, and 4) integration with primary care. These themes guided the rapid-cycle agile design process of an interface of data from an online diabetes prevention program into the primary care EHR workflow.

**Conclusions:**

The integration of external health-related data into the EHR must be embedded into the provider workflow in order to be useful to the provider and beneficial for the patient. Accomplishing this requires evaluation of that clinical workflow during software design. The development of this novel interface used rapid cycle iterative design, early involvement by providers, and usability testing methodology. This provides a framework for how to integrate external data into provider workflow in efficient and effective ways. There is now the potential to realize the importance of having this data available in the clinical setting for patient engagement and health outcomes.

## Background

Information is the currency of healthcare. With the explosion of electronic health records, that information is increasingly digitally stored and exchanged [1]. The public is digitizing personal health data faster than ever before. People regularly collect and store health-related data electronically, on their own devices and in the cloud, with direct implications for their health [2]. From steps on a pedometer to calories consumed to biometric data to personal reminders, there is more health-related data outside of healthcare systems than in it [1, 3]. Healthcare has historically relied on patients to provide this information to their providers manually, however there is an emerging opportunity to integrate this data directly into the electronic health record. To be clinically effective, however, this data must be integrated into the increasingly complex healthcare team workflow [4, 5]. Only with optimal integration of this data – the who, what, when, where, and how of human-computer interactions – will the promise of improved healthcare delivery effectiveness and efficiency from health information technology be realized.

The Health Information Technology for Economic and Clinical Health (HITECH) act mandates the ability to electronically capture and share health data, and much effort is being put into achieving this goal [6, 7]. However, there has been little focus on the usability of the data [8–14]. The most successful software systems have paid great attention to the look and feel of their product. In the same way, attention must be paid to the integration, workflow implications and visualization of data in the electronic health record (EHR). And the data does not have to just come from other healthcare systems – it can come directly from the patient or from other software systems that the patient interacts with.

It is projected that by 2030, more than 2 billion people worldwide will be overweight and 1 billion will be obese [15]. As a close corollary, by 2025 it is estimated there will be 380 million people living with type 2 diabetes [16]. In the United States alone, diabetes cost more than $200 billion in the year 2007 [17]. While the burden of disease is great, so also is the opportunity. As many as 90% of the cases of type 2 diabetes are attributable to diet and lifestyle, and thus potentially preventable [18]. Not surprisingly, a number of initiatives have been developed to target behavior change to prevent obesity and diabetes. Most notably, the Diabetes Prevention Program (DPP) and its derivatives have been largely successful in controlled studies [19–21]. But these interventions have questions of reproducibility and scalability. Likewise, consensus statements for the treatment of obesity and diabetes place an importance on exercise and diet but primary care providers are frustrated with how to counsel their patients to achieve these lofty goals [22, 23]. One potential solution has been to utilize online tools to engage patients in lifestyle modification programs. The Goal-focused Online Access to Lifestyle Support (GOALS) program is an online adaptation of the DPP that aims to curb the burden of diabetes through online education and coaching. The program incorporates physical activity tracking, diet reporting, and educational modules with personalized lifestyle coaching. Thus far, the program has been shown to help patients lose weight and improve blood pressure control [24]. Despite their successes, currently there is limited meaningful communication between providers and behavior modification programs such as DPP or GOALS. This is a lost opportunity since physician counseling has been shown to have a significant impact on patients’ weight loss and is expressed as a key motivator [25, 26]. With the optimization of electronic health records (EHRs) closing the digital divide, there now exists the opportunity to integrate updates from external lifestyle counseling seamlessly into the primary care visit using patient-generated data. However, as it stands, there remains a void in the ability of EHRs to integrate this lifestyle modification data in a useful way.

Effective design and implementation of EHR tools require studying the usability of these tools. Formal testing of these EHR features and tools with end-users has become essential to enhancing usability and outcomes, and meaningfully using EHRs [27–30]. Human computer interaction related to using the EHR has been studied using think-aloud, near live and live usability testing as well as surveys and interviews [31–36]. These use both quantitative and qualitative methodology to identify usability issues prior to adoption of the software. Agile, rapid-cycle design of the software that incorporates end-user feedback early in the design phase can decrease development time, provide transparency to the design process, and help ensure that the end-product reflects current practice patterns [37–39]. To date, little is known about the preferences of providers concerning the type, amount, and location of behavior change data or regarding its optimal workflow interface with the EHR.

Employing the use-case of the GOALS online diabetes prevention program, we performed a qualitative study to characterize the preferences of providers concerning the integration of externally generated lifestyle modification data into a primary care EHR workflow and an iterative design process to develop that interface.

## Methods

We used a qualitative study design through semi-structured interviews with primary care providers to understand their clinical workflow and preferences towards integration of external health-related data in the EHR. The study was conducted in 2 phases. Phase 1 consisted of interviewing primary care providers about their perspectives on integrating lifestyle data into the EHR and showing them a rough mockup for early feedback. Phase 2 brought the providers back for more usability evaluation with a fully developed prototype. We used rapid-cycle design methods to refine the prototype interface between each usability evaluation.

### Study setting and participants

We recruited primary care providers from the Section of General Internal Medicine and Department of Family Medicine at Boston University School of Medicine (BUSM) to participate in formative interviews. BUSM faculty’s main practices are located at Boston Medical Center (BMC), which is a large safety-net academic medical center in Boston, MA. The two adult primary care practices care for over 50,000 patients, with the vast majority coming from surrounding urban neighborhoods and having public insurance coverage.

Primary care providers were purposively selected to represent a cross-section of the faculty based on years in practice, technology avidity, change avidity, percent clinical effort, and clinical leadership role. Candidate providers were approached by study staff via email to participate in in-person interviews. They were recruited to participate in September 2014.

Providers who completed the first in-person interview were again approached in September 2015 to complete a second in-person interview to review a new version of the interface.

All interviews were recorded for later review and coding. Providers were not compensated for their time.

### Procedures

**Fig. 1:**
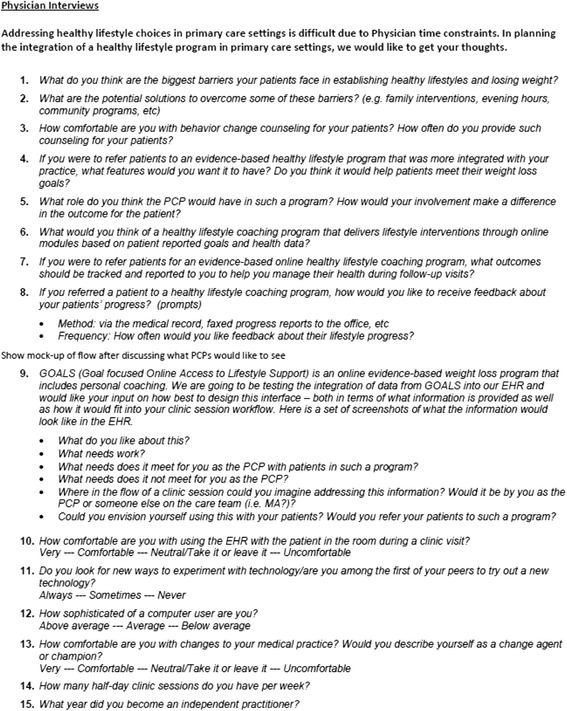
Semi-Structured Design Usability Interview Guide

**Fig. 2:**
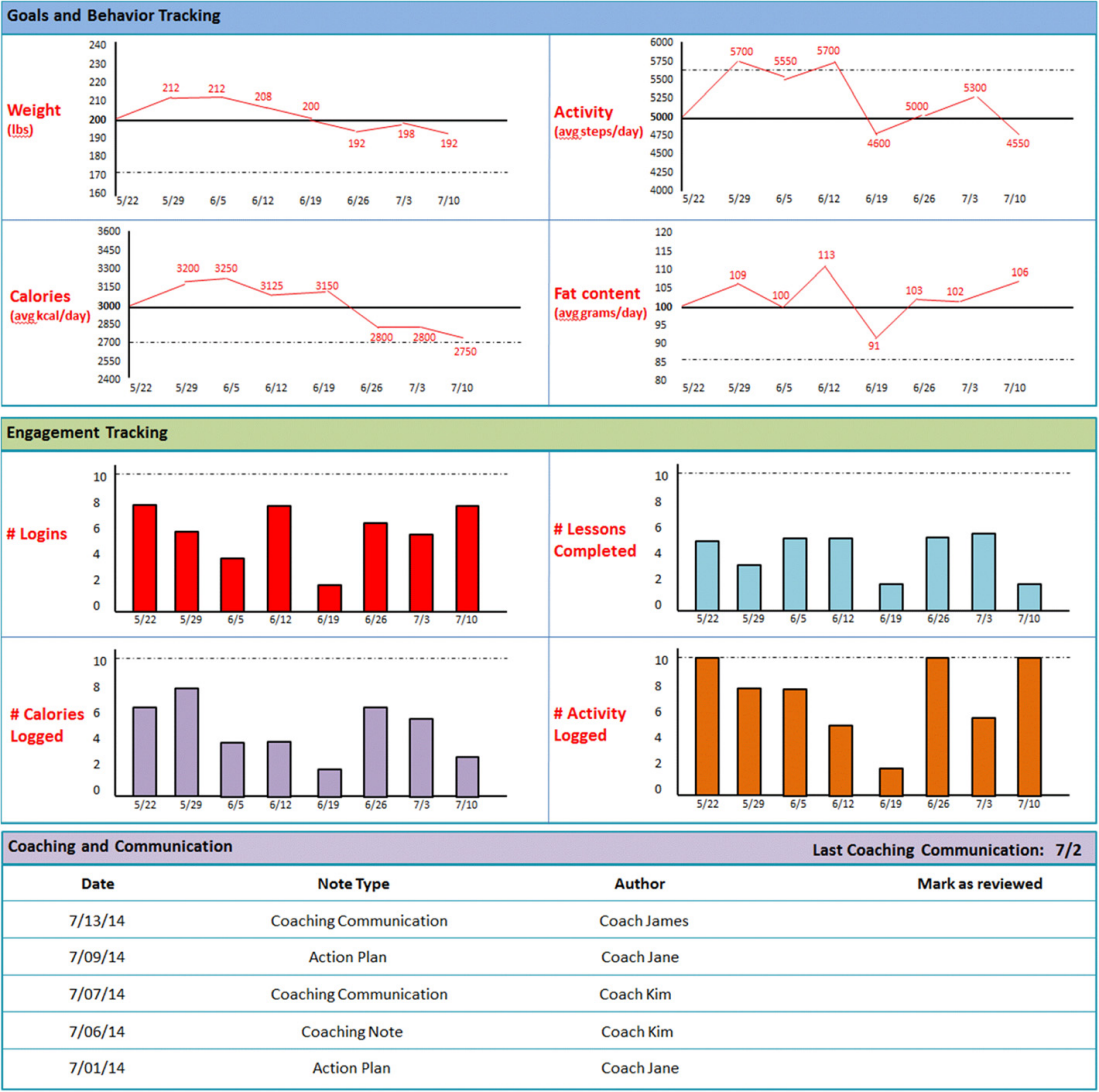
Phase 1 Usability Interview Prototype

Phase 1 Usability: Based on our goal of learning more about providers’ barriers to behavior change counseling and their desire to integrate external health-related data into their practice, we developed a semi-structured interview guide (see Fig. 1) that covered: 1) comfort with behavior change counseling, 2) understanding of lifestyle modification programs, 3) perceived patient barriers to weight loss and diabetes prevention, 4) clinical workflow, and 5) review of a potential EHR interface for lifestyle modification data. The interview included reviewing a mock-up of one potential EHR interface to view this external data (see Fig. 2). The static prototype was displayed on a wide-screen computer embedded in the EHR frame to simulate the EHR work environment of those providers. The interview concluded with demographic questions as well as questions related to the provider’s comfort with and avidity toward technology.

**Fig. 3:**
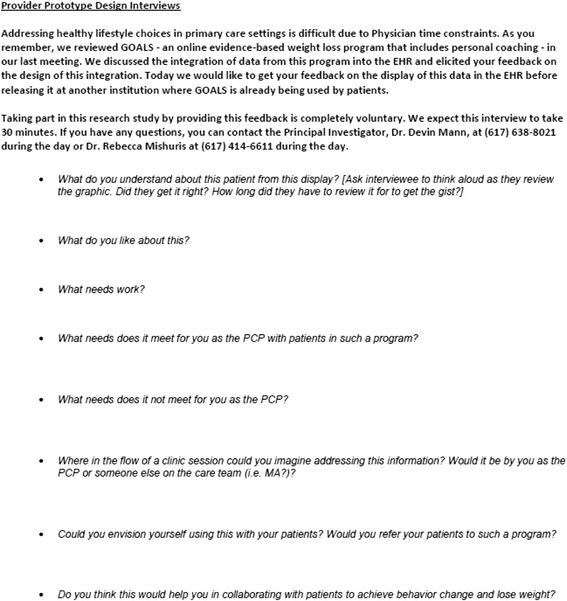
Semi-Structured Prototype Usability Interview Guide

**Fig. 4:**
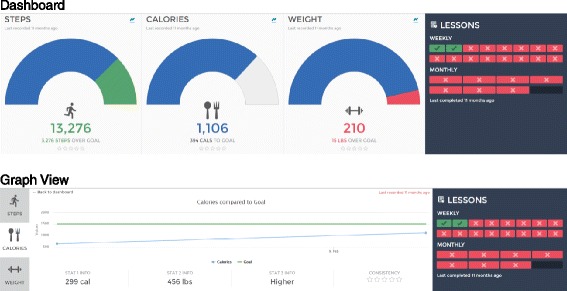
Phase 2 Usability Interview Design

Phase 2 Usability: The second round of interviews was guided by semi-structured questions (see Fig. 3) around the near-complete interface design to gain additional feedback from potential end-users on the usability of the interface (see Fig. 4). The interview was almost entirely done while allowing the provider to freely explore the interface online in an interactive setting simulating the EHR used by the providers. Data from multiple dummy patients were available for the provider to examine different potential patient scenarios. Think-aloud methodology was used to encourage the provider to narrate their actions and impressions as they interacted with the system by clicking on links and interpreting the displayed information. Interviews were recorded and written notes taken by the interviewer throughout the interview.

This study was approved by the Institutional Review Board at Boston University School of Medicine. Informed consent was verbally obtained from all interview participants.

### Analysis and rapid-cycle iteration

The study team employed grounded-theory thematic analysis of the provider interviews to identify major themes related to the design of the user interface of external health-related data into the EHR. Independently, the interviewer and another reviewer listened to the recorded interviews to identify these themes. Each reviewer recorded ideas while actively listening to the recorded interviews. The number of interviewees that mentioned each idea was tabulated. The idea lists and tabulations from each reviewer were compared; only minor discrepancies were found and these were resolved by listening to recordings again. Ideas were grouped into larger themes during iterative conversations between the two reviewers. A final list of 4 themes related to the 5 areas covered in the initial interviews was provided to the study leaders and software developers to inform the software design.

Rapid-cycle design was employed between the study leaders and the software developers through weekly meetings over a 6 month period during which design components were reviewed for visual acceptance and usability based on initial provider interviews and study leaders’ own clinical practice. The result was a series of designs culminating in the prototype shown to providers during the second round of usability interviews; after this, minor changes were made to the final design to improve provider ease of use.

## Results

### Provider interviews

Nineteen providers were approached and ten agreed to be interviewed. All who agreed, completed the first in-person interview. Eight of the ten who completed the first interview were approached to complete the second-round interview. The remaining two providers were not approached as they were no longer faculty at Boston University School of Medicine. Five of the eight providers approached, completed the second in-person interview.

Among the initial ten providers interviewed, nine practiced in General Internal Medicine and one practiced in Family Medicine. They generally represented the practices’ providers in gender and race, as well as clinical practice and comfort of use of the electronic health record (see Table 1).

**Table 1:** Interviewee characteristics

Gender	Completed Phase 1 interview only (*N* = 10)	Completed Phase 1 and Phase 2 interviews (*N* = 5)
Men	3	1
Women	7	4
Race		
Caucasian	7	5
African American	2	0
Asian American	1	0
Degree		
MD	8	4
NP	2	1
Half-day clinic sessions/week (average)	4.8 sessions	4.3 sessions
Years in practice (average)	14.6 years	14.4 years
Comfort with EHR		
Very comfortable	7	2
Comfortable	2	2
Uncomfortable	1	1
Likelihood to look for new ways to experiment with technology		
Always	2	1
Sometimes	4	2
Never	4	2
Comfort with computer usage overall		
Above average	4	2
Average	5	2
Below average	1	1
Comfort with medical practice change		
Very comfortable	4	2
Comfortable	1	1
Neutral	4	1
Uncomfortable	1	1

### Design themes

Four themes emerged during the interview (see Table 2), each is described in detail below.

**Table 2:** Design themes (*n* = 10 interviewees)

Theme	Detailed idea (number of mentions)
Barriers to establishing healthy lifestyles	• Limited access to healthy food (6) • Limited access to exercise options (6) • Health literacy (6) • Multiple competing priorities (4) • Cost (5) • Lack of time to cook well, exercise (4) • Cooking and food storage facilities, homelessness (3) • Motivation (4) • Culture/family (2)
Features of lifestyle modification program	• In-clinic contact for warm handoff (8) • Community-based program (5) • Cultural, language sensitivity (4) • Economic sensitivity (2) • Education provided pictorially (2) • Physical activity facilitation – pedometer (2) • Incentives to participate (2) • Integrate with PCP for synergy in messaging (3) • Social experience/support, peer network (1) • Help patient set small realistic goals (1) • Makes it easy to keep coming back (1) • Remote component – phone/online (1) • Provide stress management/coping skills counseling (1) • Outcome/evidence based (1) • Scalable/few resources/automated in some way (1) • Identify patient and MD goals – work toward mutual goals (1)
Reporting of outcomes to primary care provider	• Patient identified goals (5) • Patient’s level of engagement (7) • Biometric data – weight, blood pressure, hemoglobin A1c (6) • Patient’s barriers, red flags that would make success near impossible (6) • Overall sense of progress (7) • How patient relates choices to health outcomes (1) • Food group breakdown versus fat/carbohydrate breakdown (2) • Patient’s action plan (1)
Integration with primary care	• Quarterly updates (6) • Access and use in real-time with patients (7) • Provider alerts for new data (2) • Display in the EHR: graphical display, access coaching notes, patient goals and barriers (10) • Train ancillary staff to use data with patients (7) • Way to acknowledge receipt (1)

#### Barriers to establishing healthy lifestyles

Among these safety-net primary care providers, there was almost uniform agreement that patients had multiple barriers to creating and sustaining healthy lifestyles. Limited access to healthy foods and walkable neighborhoods, low health literacy, and multiple competing priorities negatively impact a patient’s ability to stay healthy regardless of their motivation.

*“There’s a systemic problem of food environment; we can do a little as primary care docs but it’s a more systemic problem”*

*“Everybody can walk but people’s neighborhoods aren’t safe…people are afraid to walk in their neighborhoods sometimes; another challenge with our patients is affordability of healthy food and place to exercise and…depression; they have so much stress in their lives that food [becomes the answer for their stress]”*

*“Some of [the barriers] is [sic] health literacy; desire is there but they just don’t understand what to do”*

Overcoming these barriers is complex, and often outside the realm of what seems possible through a primary care clinic.

*“[Patients] need a lot of guidance and hand holding and it’s hard to do that in a primary care visit”*

*“From a healthcare point of view, we’re really not set up to do that whole process [of counseling around lifestyle changes]; this is a very medical model of care”*

#### Features of lifestyle modification program

All providers identified the desire to have some in-clinic contact with the patient to initiate the online diabetes prevention program. Providers believed that this would help motivate patients to join and stay active in such a program.

*“Embedded here, would be wonderful, to have a nutritionist here on the floor to see patients before or after us [doctors]; help patients go more reliably”*

*“It’s great we’re doing population management, but it’s still on us…We have to get them while they’re here, if it was someone down the hall, it needs to be at the time of the visit to make the provider more efficient and to motivate the patient to do it and so the patient gets what they need at the time of the visit”*

Some providers wondered whether there could be a community-based aspect to the program; this would help patients stay engaged by being more culturally, socioeconomically, and geographically sensitive to patients’ needs, and by allowing patients to share resources and ideas with each other.

*“Most successful studies employed remote counseling; in-person arm didn’t do any better than the remote arm because people don’t show up for appointments; I think remote is key”*

*“Patients have resources and ideas that could be really helpful for other patients; like a buddy system or sharing in group visits”*

The importance of cultural and economic sensitivity was raised by many providers – lifestyle programs are only successful if they can meet the patient where they are and make changes within certain confines.

*“Culturally relevant cooking classes are a big resource we have”*

*“I dream of a walking group in all the relevant neighborhoods that had people who spoke the right languages”*

#### Reporting of program outcomes to the primary care provider

First and foremost, providers wanted to know the patient’s goals in the program – what were they and how were they developed. Knowing a patient’s goals would help with motivational interviewing regardless of whether the patient was on track or had fallen behind in meeting their goals.

*“Which goals were the patient working on; some sense of where the patient was at in terms of motivation and next steps”*

Providers identified the need for a sense of the patient’s engagement with the diabetes prevention program; this information was deemed essential to determining the root cause of a patient’s degree of success in meeting their goals.

*“You can look at process or outcome; if they are participating and how often they’re doing it and have we seen any results in their weight in general”*

*“The big issue is that so few of my patients are engaged in any way, I just want to know that they’re engaged, someone is checking in on them, they’re having progress forward”*

Providers also wanted a sense of the patient’s barriers to success – whether from coaching notes or from the patient themselves – to explore these further with the patient and develop plans to overcome them. Providers genuinely wanted to support their patients in their efforts in healthy living; they saw this integration as a way to augment their inadequate mechanisms currently.

*“I would love to know what’s happening so when I see the patient I can reinforce, like a warm handoff from the program; would know we were giving consistent advice and weren’t shooting each other in the foot”*

*“We know that [patients] trust [in their providers] improves some outcomes so maybe [having the primary care provider more connected] would, too”*

#### Integration with primary care

All providers thought that integrating external health-related data from between visits would help them better understand their patients and help their patients in disease prevention.

*“My role [as the primary care doctor] is to get them to go to these programs, reinforce; for patients that don’t need the whole package, I provide basics”*

*“I could seeing going to this [the integrated data display] rather than just the vital signs; looking at participation, how they’re doing against their goals, and what the next steps are; it’s more information than I have now”*

Many wanted quarterly updates on patients, but most importantly that the data be available to access and use in real-time with patients during clinic visits. Some identified the possibility of getting alerts when there was new activity from the patient in the system.

*“As soon as you log in to the chart is there a way to know that the patient is in the program…because then I could see myself wanting to use it. I can see myself wanting to use the extra information to talk with the patient”*

*“Would be nice if it was in the EMR, would connect to the [entire] patient [record]”*

*“Quarterly would be plenty; I don’t think we’re going to see results so quickly”*

Providers wanted the data in the EHR with graphical, easy to interpret displays of the biometric data as well as access to the coaching notes and identified barriers.

*“You see a goal – they’re green on weight, red on calories; easy to see quickly, to use as a teaching tool for the patient”*

*“Ideally something that you could see easily in the context of your next visit, so you don’t have to go searching for it; would be helpful if the key point was in an easy, findable spot; I’d want a summary that didn’t take more than a click to get to”*

Most wanted to use the data during a patient encounter, but most also wanted to train ancillary staff to use the interactive tool to engage with patients outside of the provider visit. Time constraints on the provider visit were a concern for all.

*“If you can slice it off, ‘I have this great program and you can talk with this person,’ laying the foundation and then having someone to follow up on that”*

*“Use this during a planned chronic care visit, but patients often save their urgent issues for their planned visits…so we never get to this”*

*“What helps people lose weight is having those reminders and someone that they’re coming back to, have some accountability. I don’t think we can underestimate our influence [as primary care providers] for a certain part of the population”*

### Iterative design process

**Fig. 5:**
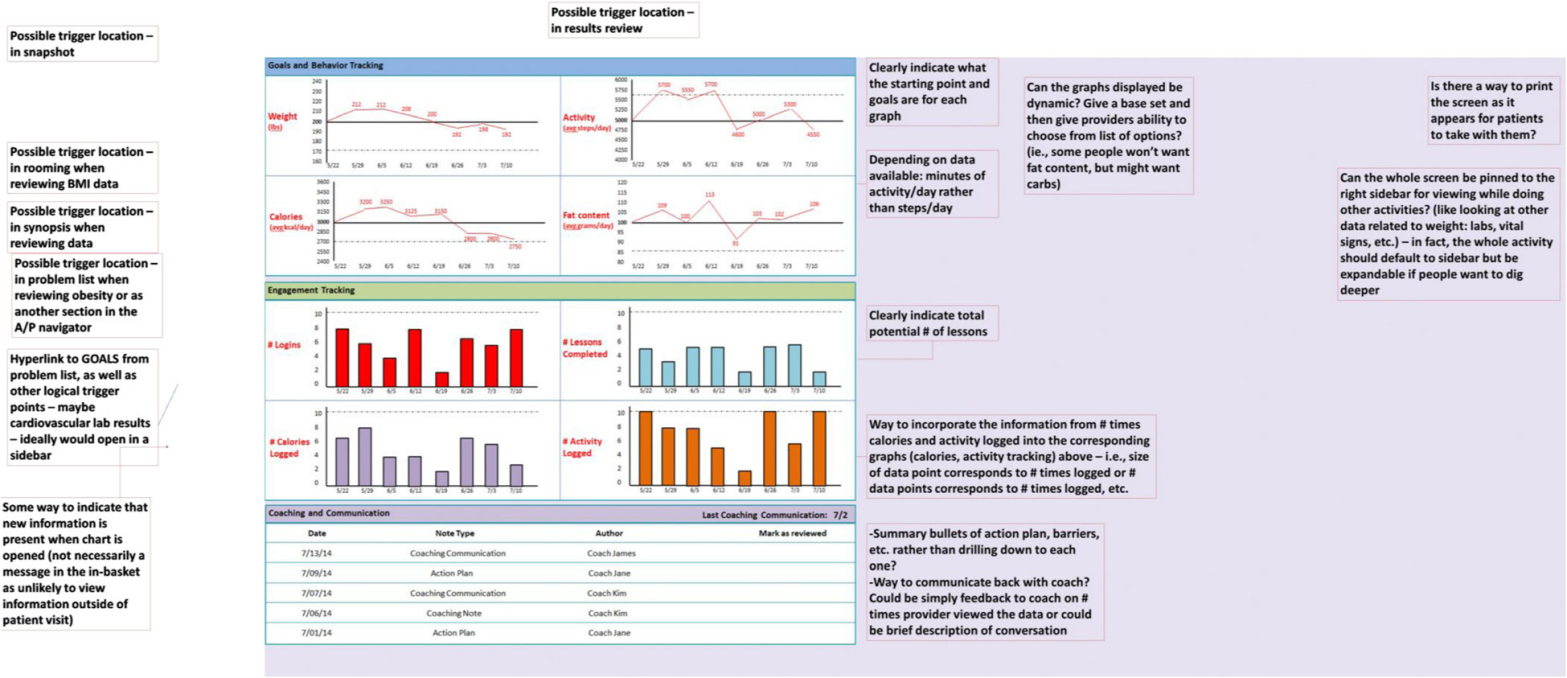
Post-Phase 1 Usability Interview Design

**Fig. 6:**
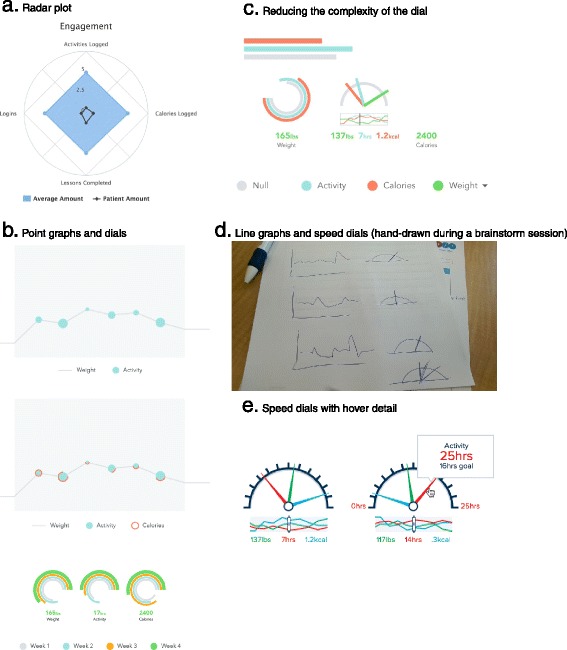
Rapid cycle iterative designs – evolution of the dashboard and data view

The study team engaged with an EHR design firm to develop the data integration and user interface prototype (see Fig. 2) that was presented to the study providers in the Phase 1 usability evaluations. The data presented in the user interface are a graphical representation of the data entered by patients into the external online diabetes prevention program GOALS. Weekly meetings allowed for rapid cycle iterative design and preliminary usability testing by study staff. Using the data collected in the Phase 1 usability sessions, a series of prototype refinements were created (see Fig. 5 for workflow, Fig. 6 for graphical display iterations, and Fig. 4 for the near-final dashboard and graphs). To improve usability and understanding of the data, the data visualization of patient-entered data from GOALS in the EHR evolved from bar charts to radar plots to speed dials over the course of the design cycles (see Fig. 6). Providers’ desire to see both patient engagement and progress towards goals drove the development of the graphs over time, dashboard, and lesson completion display. The locations in which the graphical display would appear in the EHR (see Fig. 5) were influenced by the provider interviews and known clinical workflows; 6 trigger points were identified. Given clinical workflows, there was also a desire to be notified that there was new information to review, pin the GOALS view to the side so providers could refer back and forth, and to print the information for patients (see Fig. 5).

The Phase 2 usability evaluations used think-aloud methods to review the prototype (see Fig. 4). The users generated multiple suggestions for improvements (see Table 3). For example, the initial display is a dashboard with three dials – exercise, calories, weight; most implicitly understood this, but immediately wanted greater detail and navigated to the more granular graphs with data over time in each domain. Most tried to click on the pictorial display from the dashboard to access these graphs, but this was not enabled for navigation at that time (this was a design point that was later changed). Although providers were very interested in the details of the weight, calories, and activity graphs, they also appreciated a quick view look at how those things interacted with each other and general patient engagement; this led to the presentation of a dashboard that allowed for drilling down into the specific data for each content area (see Fig. 4). Many providers did not grasp the difference in timeline between the different graphs, although this was an important point to see trends over time and relationships between the various types of data presented. All agreed that though this information would be very useful in patient discussions around healthy lifestyles, another role in clinic outside of the primary care provider might be better suited in training and time availability to review all the data with the patient.

**Table 3:** Phase 2 usability interview suggestions (*n* = 5 interviewees)

Idea (number of mentions)
Pros
Graphs – seeing the trend over time is more useful than high-level dashboard) (5) A lot of good information in graphs to use to support/guide motivational interviewing (4) Text clear/explained well/visually pleasing (3) Lessons completed give sense of engagement (2) Points on graph give sense of engagement (2) Provides more data than just from clinic visits (1)
Areas for Improvement
Where do the numbers on the dashboard come from/over what timeframe? Is it steps per day averaged over some time or just the last recorded? (4) Either highlight time axis or make consistent from graph to graph (3) Can we see high level content of lessons? What’s the difference between weekly & monthly lessons? (3) Can you show BMI along with weight? (2) Red “X” in lessons means not done or not done yet? What’s the goal number? (2) Can we view this and document our clinic visit simultaneously? (1) Can you drill down to graphs via clicking on the odometer? (1) Bar bell – thought of lifting weights but probably means pounds given what is recorded (1) Give the goal on the dashboard (rather than forcing user to do math) (1) Can you create a toggle on the graphs to show a few different intervals? Total time, 3 months, since last visit? (1) Steps can’t be less than zero – fix Y-axis so it doesn’t go negatively (1) Can you give a sense of how often data should be recorded versus what is actually recorded? (1) Give overall trend line on graphs to smooth out the noise? (1)
Use-Cases
Would use at point of care – clinic visit, phone call (5) Use by health coach/RN/MA in clinic (4) Time constraints for physician use (4) Would definitely use in patient care (motivating to review with PCP, even just having level of engagement is useful) (5)

## Discussion

Integrating health-related information into the clinical workflow is paramount in the ability to care for the entire patient; to be responsible for population health. Lifestyle modification programs are an invaluable part of preventive medicine, [40] but are often provided outside of the healthcare institution. Through semi-structured interviews, usability evaluations, and an iterative EHR interface design process, we developed an interactive widget, embedded in the providers’ usual EHR workflow, to give them seamless access to their patients' data generated in an online healthy lifestyle coaching program designed to reduce the incidence of diabetes.

Although EHRs contain a multitude of patient health data sources, they often do not easily pull in data from external systems in a clinically intuitive manner, particularly with non-traditional health data sources such as web-based lifestyle change programs [41, 42]. Patients are increasingly using online sites and mobile applications to help them get and stay healthy [43–45]. Healthcare providers have no insight into this experience or data. This novel interface provides an early experience on how to develop an interface that enables providers to easily follow patients’ progress and augment their current motivational efforts with patients. We have addressed a number of identified challenges of using externally generated health data in traditional healthcare: identifying pertinent information, integrating information into the health record, and aligning this information with the established clinical workflows [41].

The benefit of co-locating a group lifestyle modification program with primary care delivery has been shown, but this intervention lacked true integration of information from the program with primary care delivery [46]. To achieve this integration, we employed user-centered rapid prototyping as has been done in other design work related to clinician acceptance of EHR enhancements [47–51]. The work to involve the end-user in the design phase is crucial to developing interventions that seamlessly integrate with existing clinical workflows, that are usable and useful [49–51].

This study has limitations given the use of a singular site and EHR for design work. While the small number of participants limits the certainty of the observations, the size of this study is similar to other rapid design development studies [48, 52–54]. These studies note that most user generated feedback about the EHR can be elicited in design iterations with a small number of participants [48, 52–54]. Thematic saturation was reached with these participants, suggesting that additional clinician input may have resulted in a similar design. The design went through multiple iterations, and resulted in an interface that clinicians were accepting of and interested in using in practice. The design is limited by the constraints of the commercial EHR and the type of data being integrated such that not all of the clinically desired EHR workflow changes were technically feasible. The design of the data integration will be directly applicable to any site using this EHR, however. The generalizability to other clinical settings and other EHRs may be more limited, although the general principles and approaches likely span these local differences. With implementation in a live clinical environment, additional refinements may occur to more fully enable clinical workflow and integrate seamlessly with the workflows of different clinical roles.

Although we have developed an integration of externally generated health-related data into the EHR workflow, there is still important work to be done to overcome operational barriers to using this new data. Time constraints during a patient-provider visit may limit the utility of the new data to the provider. Practices may do well to think of additional roles, outside of the primary care provider, which might be better suited to using this data in patient interactions, leveraging the evolving team approach to patient care and population health [55–57]. The versatility of the design interface will allow access to the data from a variety of entry points, supporting multiple clinical roles and workflows. The planned implementation in a clinical environment will test the hypotheses that having this data interface will improve patient engagement and health outcomes.

## Conclusions

This report describes how iterative design leveraging rapid cycle usability testing approaches and involving providers, EHR designers, and health informatics researchers generated a clinically pragmatic EHR integration. This interface is sensitive to clinical workflows, aligns with clinical priorities, is intuitive and easy to use, and promises to help providers support and guide their patients. There is general consensus among study providers that having this external health-related data will help them and their patients; this has yet to be shown in a clinical setting. Studies demonstrating the value of this novel interface from an online diabetes prevention program into the EHR in diverse primary care settings will support the hypothesized importance of bringing this external data into the clinical realm for patient engagement and patient health outcomes.

## Abbreviations

DPP, Diabetes Prevention Program; EHR, electronic health record; GOALS: Goal-focused Online Access to Lifestyle Support.
